# COVID-19 Anxiety and Wellbeing at Work in Finland during 2020–2022: A 5-Wave Longitudinal Survey Study

**DOI:** 10.3390/ijerph20010680

**Published:** 2022-12-30

**Authors:** Atte Oksanen, Reetta Oksa, Magdalena Celuch, Anica Cvetkovic, Iina Savolainen

**Affiliations:** Faculty of Social Sciences, Tampere University, 33100 Tampere, Finland

**Keywords:** SARS-CoV-2, anxiety, psychological distress, work exhaustion, loneliness, self-regulation, social support

## Abstract

The COVID-19 pandemic impacted workers globally during 2020–2022 and it has had major psychological implications for workers’ wellbeing. This longitudinal study analyzed risk and protective factors predicting COVID-19 anxiety among workers in Finland. Longitudinal national sample of Finnish workers (n = 685) participated in a five-wave study conducted in 2020–2022, covering multiple waves of the COVID-19 pandemic and its aftermath. Our outcome measure was COVID-19 anxiety. Predictors were psychological distress, work exhaustion, technostress, and loneliness. Models also controlled for self-regulation; social support at work and remote working; and socio-demographic background factors. Both within-person and between-person effects were analyzed using multilevel hybrid regression models. COVID-19 anxiety varied between time points which is explained by changes in circumstances during the pandemic. Highest anxiety was expressed in the middle of the Delta variant surge and lockdown in spring 2021. Within-person changes in psychological distress, work exhaustion, technostress, self-regulation, and perceived loneliness were all associated with COVID-19 anxiety. Between-person results showed that distressed, exhausted, technostressed, and lonely workers expressed more anxiety than others. Remote workers reported higher anxiety over time than others. Those who had reported high self-regulation reported lower anxiety than others. Female gender and younger age were associated with higher anxiety. COVID-19 anxiety continues to be an important phenomenon with a magnitude of consequences on people and numerous industries. This study showed that general mental health and work stressors predict COVID-19 anxiety. Promoting social support and workers’ self-regulation skills can be beneficial for overcoming anxiety during and after the pandemic.

## 1. Introduction

The spread of the SARS-CoV-2 virus and COVID-19 disease impacted work conditions fundamentally in 2020–2022. Employees were forced to adapt rapidly to unexpected changes in ways of working and work settings which have also put a strain on the mental health of various professionals [1,2,3]. Uncertainty, fear, and anxiety characterized the pandemic as different governments were forced to take rapid actions [4,5,6]. The uncertainty faced during the pandemic was multifaceted, with many spheres of life being subjected to major sudden and unpredictable changes, including people’s work and home life. Consequently, many experienced a prolonged state of negative psychological response caused by, for instance, anxiety, worry, and fear over the COVID-19 disease and the numerous virus variants that cause it [7,8,9,10]. This involved stress about quarantine, worrying about the physical and mental health of oneself and one’s family [11,12,13,14], experiencing interruptions in vital medical treatments [15], and death anxiety [16]. According to the World Health Organization, COVID-19 death rates reached their highest point in January 2021 and since then, the death rates have decreased with occasional higher peaks [17]. The pandemic has had adverse economic and social consequences [11,12,18], and new developments are still possible.

Anxiety is a normal response under a stressful situation, such as the COVID-19 pandemic. State anxiety is an emotional state characterized by feelings of dread and tension and physiological arousal [19]. State anxiety can have both positive and negative outcomes. Even though it can motivate adaptive behaviors and coping, prolonged and severe anxiety can be especially detrimental to mental health and wellbeing [20]. A meta-analysis in the field has estimated that over 30% of people have experienced COVID-19 anxiety [8]. Past research conducted on workers shows that COVID-19 anxiety has a negative effect on their functioning in the workplace, hindering goal progression [21]. Most of the current studies addressing mental health in the workplace in the context of the coronavirus pandemic are focused on the healthcare sector. These studies indicate that healthcare workers around the globe are experiencing elevated levels of depression, anxiety, or stress that can decrease work productivity and result in absenteeism and contribute to death rates during COVID-19 [22,23,24,25,26]. Studies conducted on employees working in different industries, such as food service and lodging industry employees [27], airline staff [28], and grocery store workers [29] have yielded similar results. However, the results of these studies cannot be generalized as they are based on samples of frontline workers who were directly exposed to the SARS-CoV-2 virus in their workplace.

COVID-19 is considered an acute crisis that has similarities to large-scale catastrophes [3]. Theoretically, there is a need to understand how workers cope in such an acute crisis. Conservation of resources (COR) theory offers a framework to understand anxiety under crisis. According to COR, people seek to maintain their resources and the threat of losing one’s potential or actual resources evokes stress [30]. The COVID-19 crisis has been a continuous major stressor, and individuals’ resilience is highly dependent on the personal resources available. In terms of workers and work life, these resources could be, for example, autonomy at work, and support from supervisors and colleagues that can all foster wellbeing at work [3,31,32]. Essentially, those who have lower resources have more difficulties with gaining new resources and are more at risk of losing them [30]. Consequently, anxiety develops as stressors increase and supportive mechanisms fail [33]. Thus, those individuals might be more anxious about the situation than those who have a lot of different resources. Notably, COVID-19 anxiety has been associated with decreased COVID-19 coping and decreased general health [34].

Existing psychological problems are a major risk factor in any crisis. People with existing or increasing psychological distress are at a higher risk for experiencing COVID-19 anxiety as well [35,36,37]. Cooperation problems with colleagues, concentration problems at work, fear of getting infected at work, and health-threatening psychological or physical workload increase the risk of experiencing COVID-19 anxiety [25]. Several previous studies on the general population have found that loneliness and social isolation during the pandemic were associated with COVID-19 anxiety and psychological distress [37,38,39,40,41]. In addition, those who were concerned that they were not able to work from home during the pandemic were more likely to experience anxiety and depression [42].

Prolonged remote work increased social isolation, and it led to technostress (i.e., technology use related stress) especially during the first phase of the pandemic in spring 2020 [3]. Nevertheless remote work during the pandemic had also positive effects on workers’ wellbeing [43]. Remote work allows more freedom and flexibility in organizing the workday and managing boundaries between work and private life [43,44,45,46], but it can also cause the feeling of obligation and permanent connectivity, making it difficult to relax [47].

The presence of protective factors can help mitigate the negative impact of stressors and lower COVID-19 anxiety among workers. COVID-19 coping skills are found to buffer the negative effects of COVID-19 anxiety and support general health [34]. Social support is generally important for subjective wellbeing [48] and even more so during stressful life events [3,49]. Past studies conducted during the COVID-19 pandemic suggest that low support from supervisors is associated with anxiety and depression among workers [50] and can increase perceived pandemic-related uncertainties experienced by employees [51]. Moreover, social support has been associated with reduced remote work-related challenges and specifically alleviating work-home interface, ineffective communication, procrastination, and loneliness [52]. Another major protective factor is self-regulation. Autonomy and self-regulation are cornerstones of subjective wellbeing, according to self-determination theory [53,54]. Previous studies have associated self-efficacy with lower COVID-19 anxiety [55]. There is also reason to consider that during and after the COVID-19 pandemic, self-regulation plays an even stronger role as remote work became significantly more common and is currently an integral part of the working life, especially in certain fields like knowledge work.

Stressors of the COVID-19 pandemic have affected a large portion of the world population, but recent research indicates that there are socio-demographic differences in mental health outcomes. For instance, younger age, lower income, and female gender have been associated with higher COVID-19 anxiety [25,35,39,56]. Student status has been tied to COVID-19 anxiety, but there is conflicting research evidence on the level of education a risk factor for COVID-19 anxiety [35,57,58,59,60].

The COVID-19 pandemic provides a unique research setting to investigate wellbeing at work longitudinally. Circumstances varied a lot during the pandemic in 2020–2022, and these changing circumstances are also likely to have an impact on the perceptions people have had. For example, in Finland—the focus country of our study—the pandemic was handled very well in the first phases of the pandemic and mortality rate remained low [5]. A German magazine Der Spiegel reported in 2021 that Finland had coped the best with COVID-19 in comparison with 154 other countries [61]. In 2022, the mortality rate for COVID-19 was much higher due to the spread of the omicron variants and return to normality [62]. However, currently there are major gaps in research and the work conditions of many workers have indefinitely changed due to the pandemic. Increase in remote work is one such factor. The evidence of COVID-19 anxiety among workers is still building, and more longitudinal perspectives are needed. This longitudinal study is responding to this research gap by analyzing risk and protective factors of COVID-19 anxiety among workers, as well as by providing practical implications to support mental wellbeing at work.

This longitudinal study analyzed risk and protective factors predicting COVID-19 anxiety among workers in Finland in 2020–2022. Building on COR theory and research on stress factors under COVID-19, we hypothesized that risk factors for mental health and occupational wellbeing predict higher COVID-19 anxiety over time (H1) and protective factors at work predict lower COVID-19 anxiety over time (H2). Additional analyses aimed to show whether any of these effects were stronger at a certain time point.

## 2. Method

### 2.1. Participants and Procedure

Participants of this study included employees from mainland Finland. Participants took part in the longitudinal Social Media at Work in Finland Survey study, which targeted the Finnish working population. The response rate for the baseline survey collected in March–April 2019 was 28.31% [63,64]. Measures related to the COVID-19 pandemic were included in the survey collected during September–October 2020 (time point 1 [T1] for this study, n = 1152). Follow-up surveys of the same respondents were collected in March–April 2021 [T2, n = 1018], September–October 2021 [T3, n = 982], March–April 2022 [T4, n = 932], and September–October 2022 [T5, n = 921]. Out of the participants taking part in T1, 59% (n = 685) took part in all five time points of the study. Those participants who had completely stopped working (e.g., retired and not working at all) were omitted from analyses (n = 29). The full data hence included 3092 observations from 656 participants.

Participants included in the study were 41.62% female and aged 20 to 66 (mean age 46.07, standard deviation 10.60). Out of the participants, 47.45% were married or in a registered relationship, 22.71% had a university degree, and 11.28% had a high individual income (at least 5000 Euros per month) at T1. Out of the participants, 14.63% were working in the social and welfare sector, 13.72% in raw materials and manufacturing, 5.34% in construction, 11.13% in retail and transportation, 16.16% in the service sector, 14.33% in business, communication and technology, 7.93% in public administration, 9.45% in education, and 7.32% in other sectors. Geographically, they came from all the regions of mainland Finland: 37.96% from the Helsinki–Uusimaa area, 20.43% from Southern Finland, 23.78% from Western Finland, and 17.84% from Eastern and Northern Finland. Sample characteristics generally match the working population in Finland, and no major biases have been found during the longitudinal study. Drop-out analysis has shown that participants responding to all survey waves are slightly older, include more males, and have a higher education than the average working population in Finland according to the official statistics of Finland [40]. Analytical weights were used to correct biases of the sample.

The Academic Ethics Committee of the Tampere region in Finland stated that the study does not pose ethical problems (decision number 90/2018). Participation was voluntary. Participants were informed of the study’s purpose, and they gave their consent for participation. Data collection was administrated by Norstat, and all respondents answered the survey online. Participants were drawn from Norstat’s web-based panel. Data included only respondents who completed the whole survey. Separate data integrity checks involving attention checks, patterned responses, and nonsensical responses checks were conducted for each time point. Open-ended comments were also checked to further evaluate possible biased motives in response patterns.

### 2.2. Measures

COVID-19 anxiety was measured using a scale based on the 6-item Spielberger State–Trait Anxiety Inventory, STAI-6 [65]. The measure has been previously validated for research use [37]. Respondents were asked to assess their feelings about the COVID-19 crisis during the past seven days with six statements (e.g., “I feel tense”). The response scale for each statement ranged from 1 (does not describe my state at all) to 7 (describes my state completely). Three of the statements were reversed for the analysis. The scale had good internal consistency based on McDonald’s omega at all time points (T1: ω = 0.88; T2: ω = 0.89; T3: ω = 0.89; T4: ω = 0.89; T5: ω = 0.90; see Table 1 for details).

Psychological distress was measured using the 12-item General Health Questionnaire to assess psychological distress at each time point [66]. The measure has been validated and widely used in the Finnish context [67,68]. The answer options ranged from 0 to 3. All 12 items were summed to create a composite variable ranging from 0 to 36, with higher values indicating higher psychological distress. Internal consistency of the scale was excellent at all time points (T1: ω = 0.91, T2: ω = 0.91, T3: ω = 0.92; T4: ω = 0.93; T5: ω = 0.93).

Work exhaustion was measured using the five-item exhaustion subscale of Maslach Burnout Indicator [69]. The scale has been validated and used widely in Finnish studies on occupational wellbeing [3]. By summing the five items with answer options ranging from 0 (never) to 6 (daily), we created a scale with a range from 0 to 30. Internal consistency of the scale was excellent at all time points (T1: ω = 0.93, T2: ω = 0.94, T3: ω = 0.93; T4: ω = 0.93; T5: ω = 0.94).

Technostress measure was based on six items from techno-overload and techno-invasion subscales of Ragu-Nathan and collagues’ technostress scale [70]. Items were adapted for the purpose of our study focusing on social media, for example “I am forced to do more work than I can handle due to social media.” The scale has been validated and used in prior studies on wellbeing at work [3]. The answer options ranged from 1 (disagree completely) to 7 (agree completely), so the final scale had a range of 6–42. Internal consistency of the scale was excellent at all time points (T1: ω = 0.92, T2: ω = 0.91, T3: ω = 0.91; T4: ω = 0.92; T5: ω = 0.92).

Self-regulation was measured with a 2-item scale based on the self-determination theory [53,54]. Items were “I am able to independently regulate my use of time to achieve goals in my work.” and “I am able to independently regulate my ways of working to achieve goals in my work.”. The answer options ranged from 1 (disagree completely) to 7 (agree completely), so the final scale had a range of 2–14. Items had high correlation at each time point (T1: r = 0.79; T2, r = 0.81, T3: r = 0.83; T4: r = 0.82; T5: r = 0.80).

Loneliness was measured with a three-item loneliness scale adapted from the standard Revised UCLA Loneliness Scale (R-UCLA) [71]. The scale has been widely used in Finland in different general population samples [40,72]. The scale includes three statements about perceived loneliness (e.g., “How often do you feel isolated from others?”). Answer options were 0 (almost never), 1 (sometimes), or 2 (often). Higher scores indicate higher levels of perceived loneliness. Internal consistency of the scale was good at all time points (T1: ω = 0.84, T2: ω = 0.85, T3: ω = 0.86; T4: ω = 0.84; T5: ω = 0.85).

Social support at work was measured with four questions on supportive working environment and support received from colleagues and supervisors. The items derived from the Copenhagen Psychosocial Questionnaire (CPSQII) [73]. The scale has been used in prior studies in Finland [63]. The scale had a range from 4 to 20. Internal consistency of the scale was excellent at all time points (T1: ω = 0.79, T2: ω = 0.82, T3: ω = 0.81; T4: ω = 0.81; T5: ω = 0.80).

Remote work was measured at each time point with questions on whether respondents had worked remotely and how often they worked remotely. For our analyses, we created a dummy variable showing those working remotely at least three days a week.

Socio-demographic and occupational information were used as controls. These included age, gender, marital status, education, occupational area, and income. For education level, we used a dummy variable categorizing those having a university degree (22.71% at T1). Those working in the social and welfare sector (14.63% at T1), with an income of at least 5000 euros per month (11.28% at T1), and married (47.45% at T1) were also dichotomized.

### 2.3. Statistical Modelling

All analyses were conducted using Stata 17 software. Descriptive results of the study are reported in Table 1 and the text. We provide information about general changes in COVID-19 anxiety during 2020–2022. Statistical significances between time points are based on multilevel fixed effects (within-person) regression.

We ran the main analyses on longitudinal predictors of COVID-19 anxiety using linear multilevel hybrid models. Hybrid models enable simultaneous estimation of within-person effects and between-person effects. Such models combine the strengths of random-effects and fixed-effects approaches and solve their shortcomings [74,75]. For the analysis of hybrid models, we used xthybrid command in Stata [75].

All our main time-varying variables had both within-person and between-person effects. We first ran zero models (model 0) by including only one independent variable while adjusting for age and gender. The full model included all main variables and control variables in the same model. All variables were standardized for the regression models. Models report regression coefficients (B) and their robust standard errors (SE). We also report 95% confidence intervals (95% CI) of B, Z-statistics, and *p*-values for statistical significance. Within-person effects show how changes over time in predictors are associated with the change in the outcome variable. Between-person variables show group differences between individuals. The final model also included between-person control variables.

The last part of the analysis involved checking interactions with time points to see whether some effects of the key within person effects were stronger during different time points. These fixed effects multilevel models were run separately for all main independent variables. Also, the directionality of effects was checked separately with standardized variables and the effects were significantly stronger when independent variables were predicting COVID-19 anxiety than vice versa. The only exception was self-regulation which had a slightly bigger effect when COVID-19 anxiety was predicting it.

## 3. Results

**Figure 1:**
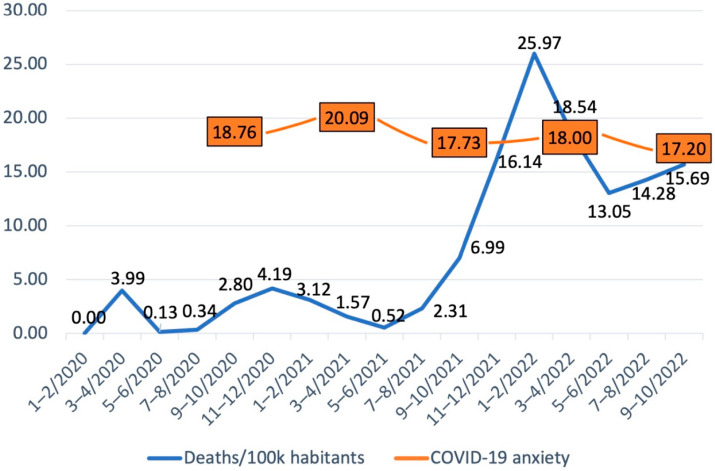
COVID-19 mortality and COVID-19 anxiety in 2020–2022. Note. Figure 1 COVID-19 mortality figures are based on the information reported by the Finnish Institute of Health and Welfare [62].

The results showed that anxiety was highest at T2, representing spring 2021 (see Table 1 and Figure 1). Within-person analysis showed that both the increase of anxiety between T1 and T2 and decrease of anxiety between T2 and T3 and between T4 and T5 were statistically significant (*p* < 0.001). Figure 1 shows the average level of COVID-19 anxiety and COVID-19 related deaths per 100 000 inhabitants over time. At T1, COVID-19 anxiety was correlated with higher psychological distress, work exhaustion, technostress and loneliness, and lower self-regulation and social support at work (*p* < 0.001, see Table 2).

Hybrid models reported in Table 3 show results based on models that are adjusted only by age and gender, each independent variable having its own model (model 0). We found that all our main independent variables except remote working had statistically significant within-person effects. Psychological distress had the strongest within-person effect out of the variables (Z = 9.53, *p* < 0.001) indicating that an increase in psychological distress is associated with an increase in COVID-19 anxiety. Work exhaustion and perceived loneliness also had relatively strong effects. Between-person effects of the same variables were all statistically significant, except for remote work. Psychological distress had the strongest between-person association with COVID-19 anxiety.

The full model included all the variables and control variables in the same model. The results showed statistically significant within-person effects: an increase in psychological distress, technostress, and perceived loneliness were associated with an increase in COVID-19 anxiety. An increase in self-regulation was associated with a decrease in COVID-19 anxiety. A within-person effect of psychological distress was strongest out of all risk and protective factors included in the model (Z = 7.61, *p* < 0.001). The between person-effects part of the model showed that those reporting higher psychological distress, work exhaustion, technostress, and loneliness had a higher score in COVID-19 anxiety. Higher self-regulation was associated with lower COVID-19 anxiety. Remote workers, women, and younger people reported higher levels of anxiety. The last part of the analysis investigated whether any of the within-person effects were stronger at certain time points. We found no statistically significant differences in this analysis.

## 4. Discussion

This longitudinal study based on data collected in five time points during 2020–2022 examined the risk and protective factors predicting COVID-19 anxiety among workers. We based our work on the Conservation of Resources Theory [30] and found support for our hypotheses. Psychological distress, technostress at work, and perceived loneliness had within-person effects on COVID-19 anxiety. In other words, as these risk factors increased, so did COVID-19 anxiety. These results underline how crises like the COVID-19 pandemic can lead to resource loss and impaired coping, thus aligning with the COR theory [30]. We also found that self-regulation functioned as a protective factor, which highlights the importance of individuals’ existing resources as a buffering element in aversive events like pandemics. All these risk and protective factors also had between-person effects indicating the differences between individuals. Work exhaustion had a between-person, but not a within-person effect and was statistically significant only in the first model adjusted for age and gender. Prior literature has indicated that wellbeing has stayed rather stable, and burnout and work exhaustion have not changed considerably during COVID-19, depending on the individual factors examined [3,76,77]. Finally, remote work did not have a within-person effect on COVID-19 anxiety, but those who worked remotely at least three days a week reported higher COVID-19 anxiety than those who did not, adding to the existing literature that has highlighted the negative psychological implications of remote work, such as stress [3,43].

Previous research has established that those working in operations and services industries that are considered essential (e.g., grocery and utility workers) and those working in the frontline of the pandemic (i.e., healthcare workers) experience higher levels of COVID-19 anxiety and other mental health consequences [22,23,24,25,29]. Our results add to these findings and bring new insight indicating that remote workers also show increased levels of COVID-19 anxiety over time. Although employees working from home are relatively safe from COVID-19 infection, they are still affected by the adverse consequences of the pandemic. In prior research, remote work has been associated with both positive and negative wellbeing consequences [42,44]. Many remote workers experience loneliness and technostress [3,44] which, according to this study, are significant risk-factors for COVID-19 anxiety.

Overall, our results underline that stressors at work and life in general have led to anxiety during uncertain times of the COVID-19 pandemic. Moreover, this effect on anxiety seems to hold for some workers, even though the pandemic itself has become largely accepted and treated more as an endemic toward the end of 2022. This is also shown in our results with declining COVID-19 anxiety rates despite the high mortality rate in 2022. However, given its exhaustive aftermath and new potential variants, COVID-19 can continue to cause anxiety and mental health consequences among people worldwide.

Our results suggest the existence of protective factors that can help protect workers from experiencing COVID-19 anxiety. Higher self-regulation was associated with lower COVID-19 anxiety, which is in line with previous results regarding positive outcomes of self-efficacy in relation to COVID-19 anxiety [55]. Moreover, we found some evidence that lower social support was associated with higher COVID-19 anxiety which is in line with prior findings [41,50]. Especially regarding subjective wellbeing during stressful times, social support has been found to be an important protective factor [25,44,45]. Moreover, according to past research, workers who felt safe at work and felt protected by their employers demonstrated lower levels of exhaustion [29].

Our results show that women and younger employees report stronger COVID-19 anxiety. These results are in line with findings from past research [24,28,35]. In our study, women reported higher COVID-19 anxiety. Also, prior studies have indicated that female employees show slightly higher levels of stress than their male colleagues [28]. These findings are partially explained by the employment sector as women represent a large percentage of workers in industries most heavily impacted by the pandemic (e.g., healthcare and the service industry). Furthermore, female employees working from home often attend to childcare and housework during the workday [56]. It appears that the COVID-19 pandemic has further highlighted gender inequalities, leading to high levels of exhaustion among remotely working women who have children [29]. In addition to our study showing that younger employees experience higher anxiety, prior studies have indicated that younger employees demonstrate higher levels of exhaustion [29]. It is possible that younger employees feel more pressure to excel at work and are then more impacted by the uncertainty brought by COVID-19. They might also worry more, for example, over job loss because of the pandemic [56]. Older employees, on the other hand, might have felt more secure in their positions at work or, alternatively, contemplated leaving the workforce as a form of self-care or new opportunity [78]. However, it should be noted that recent studies have identified older age as a risk factor for job insecurity and job loss, as well as negative mental health outcomes due to the pandemic [79,80].

Our results provide essential insights for organizations regarding work life during pandemics and these findings can be used to develop strategies to cope with COVID-19 anxiety in workplaces. Employees’ psychological distress, work exhaustion, technostress, and perceived loneliness are important factors to be considered when finding ways to relieve COVID-19 anxiety. Fostering opportunities for social support and self-regulation is essential for sustaining resilience and mental wellbeing and alleviating the anxiety of employees during unpredictable, stressful times of pandemics. This can mean creating employee assistance programs or simply organizing a dedicated space and time in the online working sphere for social interaction and encouraging workers to reach out to their supervisors or designated human resources specialists if they need support. Workers’ self-regulation skills can be promoted by allowing them to influence their tasks and organization of their work time when possible. Educating employees to deal with the challenges that stem from remote work and technology use at work in general, can help raise awareness and protect them from the potential negative consequences, such as technostress. Special attention should be given to women and young workers as it is shown that they are more likely to suffer from anxiety during the coronavirus pandemic.

## 5. Limitations and Strengths

Our study was limited to workers in Finland and other studies should continue to investigate COVID-19 anxiety as reactions to COVID-19 have varied quite a lot between European countries alone. Future studies should investigate the observed associations in cross-cultural contexts. We are also limited by self-reported measures. Although our focus was on risk and protective factors of COVID-anxiety, we do recognize that COVID-19 anxiety itself could have an impact on our predictors. This was most obvious with the case of COVID-19 anxiety having a larger effect on self-regulation than vice versa. Future studies could also investigate how COVID-19 anxiety impacts well-being in general. Furthermore, our study did not inquire the respondents about their perceived vulnerability to COVID-19 which could have been expected to relate to COVID-19 anxiety. Future research should consider this measure. Lastly, our analyses are based on workers between ages 20 to 66. Age is an important factor in the mental health outcomes of pandemics, and older adults were the most vulnerable age group to COVID-19 infection. Anxiety among older adults should be analyzed in future studies. We also recommend future longitudinal studies to include additional relevant predictors of anxiety, such as comorbidities, pre-existing mental health or psychological disorders, and COVID-19 infection. Despite these limitations, the current study also has significant strengths. The primary one is its longitudinal design with repeated measurements during various points of the COVID-19 crisis.

## 6. Conclusions

The COVID-19 pandemic continued longer than expected as new variants of the virus emerged and increased the number of cases periodically. At the same time, the pandemic changed the everyday lives of people and permanently reshaped the world. Under these circumstances, the pandemic may still cause anxiety. Considering the high likelihood of similar pandemics and subsequent events in the future, it is of the utmost importance to support workers’ mental wellbeing. Our longitudinal study found that stressors in work and private life can contribute to COVID-19 anxiety as it tends to increase with other added stressors. The risk factors for COVID-19 anxiety identified in this study included psychological distress, work exhaustion, technostress at work, perceived loneliness, and remote work. Self-regulation served as a protective factor, and it is increasingly important as remote work has become commonplace in many occupational sectors. These results suggest that stressors at work and life in general make adapting to prolonged crises, like the COVID-19 pandemic, more difficult. Efforts to promote self-regulation and social support at work are needed to protect workers’ mental wellbeing.

## Figures and Tables

**Table 1:** Descriptive statistics of the main study variables.

	Range	T1, M (SD)	T2, M (SD)	T3, M (SD)	T4, M (SD)	T5, M (SD)
1 COVID-19 anxiety	6–42	18.76 (6.86)	20.09 (7.15)	17.73 (6.90)	18.00 (6.85)	17.20 (7.50)
2 Psychological distress	0–36	12.08 (5.50)	12.26 (5.52)	12.29 (5.67)	12.47 (5.91)	12.46 (6.13)
3 Work exhaustion	0–30	14.51 (7.72)	14.13 (7.77)	14.30 (7.71)	14.07 (7.72)	14.52 (7.92)
4 Technostress at work	6–42	13.48 (7.79)	12.87 (7.14)	12.78 (7.34)	12.66 (7.41)	12.47 (7.27)
5 Self-regulation	2–14	9.85 (2.73)	9.87 (2.65)	9.95 (2.71)	9.85 (2.72)	9.92 (2.64)
6 Perceived loneliness	0–6	1.67 (1.60)	1.81 (1.72)	1.70 (1.68)	1.69 (1.65)	1.66 (1.63)
7 Social support at work	4–20	14.69 (3.04)	14.61 (3.10)	14.78 (3.00)	14.79 (3.03)	14.62 (3.12)
8 Remote work	0–1	0.21 (0.41)	0.30 (0.46)	0.24 (0.43)	0.22 (0.42)	0.19 (0.39)

Note. analytical weights were used. M = mean. SD = standard deviation.

**Table 2:** Zero-order correlations at T1.

Variables	1	2	3	4	5	6	7
1 COVID-19 anxiety	1						
2 Psychological distress	0.44 ***	1					
3 Work exhaustion	0.37 ***	0.54 ***	1				
4 Technostress at work	0.30 ***	0.27 ***	0.31 ***	1			
5 Self-regulation	−0.28 ***	−0.24 ***	−0.31 ***	−0.03	1		
6 Perceived loneliness	0.38 ***	0.54 ***	0.37 ***	0.16 ***	−0.15 ***	1	
7 Social support at work	−0.23 ***	−0.37 ***	−0.30 ***	−0.08 *	0.18 ***	−0.34 ***	1
8 Remote work	−0.01	−0.001	−0.07	0.02	0.18 ***	0.04	−0.002

Note. *** *p* < 0.001, * *p* < 0.05.

**Table 3:** Hybrid models showing within-person and between-person effects on COVID-19 anxiety.

	Model 0 (Age & Gender Adjusted)						Full Model					
Within-Person Effects	B	SE (B)	95%	CI	Z	*p*	B	SE (B)	95%	CI	Z	*p*
Psychological distress	1.94	0.20	1.54	2.34	9.53	<0.001	1.51	0.20	1.12	1.90	7.61	<0.001
Work exhaustion	1.18	0.22	0.75	1.61	5.41	<0.001	0.40	0.21	−0.02	0.81	1.88	0.060
Technostress at work	0.67	0.21	0.25	1.08	3.16	0.002	0.57	0.21	0.17	0.98	2.78	0.005
Self-regulation	−0.56	0.18	−0.91	−0.21	−3.13	0.002	−0.43	0.17	−0.76	−0.09	−2.51	0.012
Perceived loneliness	1.53	0.23	1.07	1.991	6.53	<0.001	0.96	0.21	0.54	1.38	4.48	<0.001
Social support at work	−0.81	0.19	−1.19	−0.44	−4.3	<0.001	−0.34	0.18	−0.70	0.02	−1.83	0.068
Remote work	0.10	0.20	-0.29	0.483	0.49	0.627	0.04	0.21	−0.37	0.44	0.19	0.853
**Between-person effects**												
Psychological distress	3.62	0.30	3.03	4.20	12.13	<0.001	1.83	0.41	1.02	2.64	4.41	<0.001
Work exhaustion	2.73	0.24	2.25	3.21	11.20	<0.001	0.58	0.26	0.06	1.09	2.17	0.030
Technostress at work	2.21	0.24	1.73	2.69	9.04	<0.001	1.29	0.23	0.84	1.74	5.60	<0.001
Self-regulation	−2.15	0.30	−2.73	−1.57	−7.29	<0.001	−1.15	0.26	−1.65	−0.64	−4.47	<0.001
Perceived loneliness	2.61	0.25	2.11	3.10	10.36	<0.001	0.95	0.26	0.43	1.46	3.62	<0.001
Social support at work	−1.85	0.24	−2.33	−1.38	−7.64	<0.001	−0.21	0.23	−0.66	0.24	−0.91	0.361
Remote work	0.37	0.22	-0.07	0.81	1.63	0.103	0.43	0.20	0.05	0.82	2.22	0.026
**Controls**												
Female	-	-	-	-	-	-	0.46	0.19	0.10	0.83	2.48	0.013
Age	-	-	-	-	-	-	−0.40	0.18	−0.76	−0.05	−2.22	0.026
Married	-	-	-	-	-	-	0.29	0.18	−0.07	0.65	1.60	0.110
University degree	-	-	-	-	-	-	0.04	0.18	−0.32	0.39	0.20	0.843
Social & welfare sector	-	-	-	-	-	-	0.17	0.19	−0.20	0.54	0.91	0.363
High income	-	-	-	-	-	-	−0.05	0.14	−0.33	0.22	−0.37	0.709

Note. All independent measures are standardized in models. Model 0 includes 7 different models that all adjust only age and gender. All models include in total 3092 observations from 656 participants.

## Data Availability

Data is available from the corresponding author with a reasonable request. After the research project, data will be made publicly available in the Finnish Social Science Data Archive (https://www.fsd.tuni.fi/en/).
